# Precipitation data in Seoul, Korea during 1778–1907

**DOI:** 10.1038/s41597-024-03335-8

**Published:** 2024-05-31

**Authors:** Jae-Won Lee, Ho-Jeong Shin, Jinkyu Hong

**Affiliations:** Ecosystem-Atmosphere Process Lab, Department of Atmospheric Sciences, Yonsei University,

**Keywords:** Hydrology, Climate sciences

## Abstract

Precipitation plays a crucial role in the global energy and water cycle and has important implications for food, water, and energy security. To enhance our understanding of the water cycle, it is invaluable to have a comprehensive historical record of precipitation. However, obtaining such records, especially for the period before the Industrial Revolution, can be challenging. During the Joseon Dynasty, Korea established a network for measuring rainfall and recorded this information in historical documents known as Seungjeongwon Ilgi and Ilseongnok. Recently, these documents have been digitized, providing us with daily precipitation data for Seoul spanning 130 years, from 1778 to 1907. By combining and analyzing these two documents, we were able to address inconsistencies found in previous studies and improve the quality of the data. Notably, this dataset is free of any missing values, making it the longest daily precipitation record in the world before the Industrial Revolution. Its availability to the public holds great potential for climate research in East Asia during the late Little Ice Age.

## Background & Summary

Precipitation plays a crucial role in the global energy and water cycle, impacting food, water, and energy security. With a changing climate, we are increasingly concerned about the socio-economic damages caused by extreme rainfall events and droughts. Historical records of rainfall are essential not only for understanding natural climate variability, but for improving flood and drought forecasts in future climate conditions^1^. Acquiring accurate and comprehensive long-term precipitation data is challenging, especially prior to the Industrial Revolution. While the surface air temperature record began in the 1660 s in central England^2^, standardized rain gauges were not introduced until the mid-18th century. Reconstructed monthly precipitation in Europe based on the diaries of observers in the 1760 s, which led to the conceptual design of the reconstruction procedure^3^. Efforts have been made to reconstruct precipitation using stable isotopes from sources such as tree rings^4^, pollen^5^, ice cores^6^, corals^7^, caves^8^, and stalagmites^9^. However, the absence of continuous precipitation measurements before the Industrial Revolution has hindered our understanding of past climate variability, particularly in relation to precipitation patterns during events like the Little Ice Age.

The Joseon Dynasty (1392–1910) in Korea established a network for measuring rainfall by inventing a rainfall gauge. This was done to promote stable agricultural productivity. Before the invention of the rainfall gauge, the amount of precipitation was estimated indirectly by measuring the depth of wet soil after rainfall events^10^. King Sejong and a group of scholars invented the rainfall gauge, known as Cheugugi (or Chukwookee), in 1441. This standardized the measurement of rainfall, which was important for rice cultivation^11^. In 1442, a plan was implemented to expand rainfall observation to other provinces in addition to the observation at the Seoul royal palace^12^. The practice of rainfall observation continued even after King Sejong’s reign, becoming a national affair.

The Cheugugi measurements recorded important rainfall information, such as the total depth of rainfall and the starting and ending times of rainfall events. The Korean government declared Cheugugi a national treasure in 2020. However, the Cheugugi rainfall records in Korea faced intermittent destruction during major wars (such as the Japanese invasion of Korea in 1592, the Manchu invasions of Korea, and the civil wars in the 1620 s) as well as occasional fire incidents until the late 18th century. In 1770, King Yeongjo restored the rainfall observation system and resumed making daily records of rainfall, including the starting and ending times of rainfall events^13^). These rainfall measurements continued at the royal palace in Seoul for approximately 130 years until the Cheugugi was replaced by a modern rain gauge in 1907.

Alongside the rainfall measurements, the Joseon Dynasty documented related information on flood events, contextualized within their political and social contexts, and the decision-making processes. These valuable records are found in three ancient documents: the annals of the Joseon Dynasty, known as ‘Sillok’ (1392–1910)^14^, the diaries of the Royal secretariat from the 17th century, known as ‘Seungjeongwon Ilgi’ (1623–1910)^15^, and a diary-style log detailing the daily lives of the Kings, named ‘Ilseongnok’ (1760–1910)^16^. These documents were recognized by UNESCO as the Memory of the World in 1997, 2001, and 2011, respectively. They provide valuable historical information on precipitation in East Asia during the Late Little Ice Age.

Several studies have been conducted to extract rainfall information from historical documents to analyze past climates. These studies have reported significant dry spells and wet periods during the late Joseon Dynasty^17–26^. However, previous efforts had limitations as they relied on manually deciphering characters from image files and lacked extensive quality check procedures for rainfall observations. These limitations can be overcome with the recent digitization of historical documents by the Korean government, including ‘Seungjeongwon Ilgi’ and ‘Ilseongnok’. This digitization allows for the accurate extraction and search of precipitation records, minimizing potential errors associated with visually interpreting Literary Chinese in which the historical documents were written. Furthermore, it facilitates easy verification and consistency checks among different documents.

This study focuses on reconstructing precipitation data in Seoul, Korea from 1778 to 1907 by parsing and compiling the digitized records of ‘Seungjeongwon Ilgi’ and ‘Ilseongnok’. Thorough quality assurance processes have been conducted. Our dataset is particularly reliable compared to previous studies due to our efforts to minimize manual reading errors and address missing values. By cross-checking the two aforementioned documents, we also ensured consistency, eliminated redundant information, and achieved a complete data time series without any missing values. The detailed methodologies used for retrieving the precipitation records and the quality assurance processes are described with examples for future studies.

The significance of this study lies in its provision of exclusive daily precipitation data from the late 18th and 19th centuries in a mid-latitude region, offering valuable insights into past regional climate research in East Asia. The reconstructed precipitation data in this study is particularly relevant to densely populated areas where the timing and quantity of summer monsoon rainfall have significant influences on food and the environment^27–30^.

## Methods

### Conversion of units, time, and calendar

**Fig. 1:**
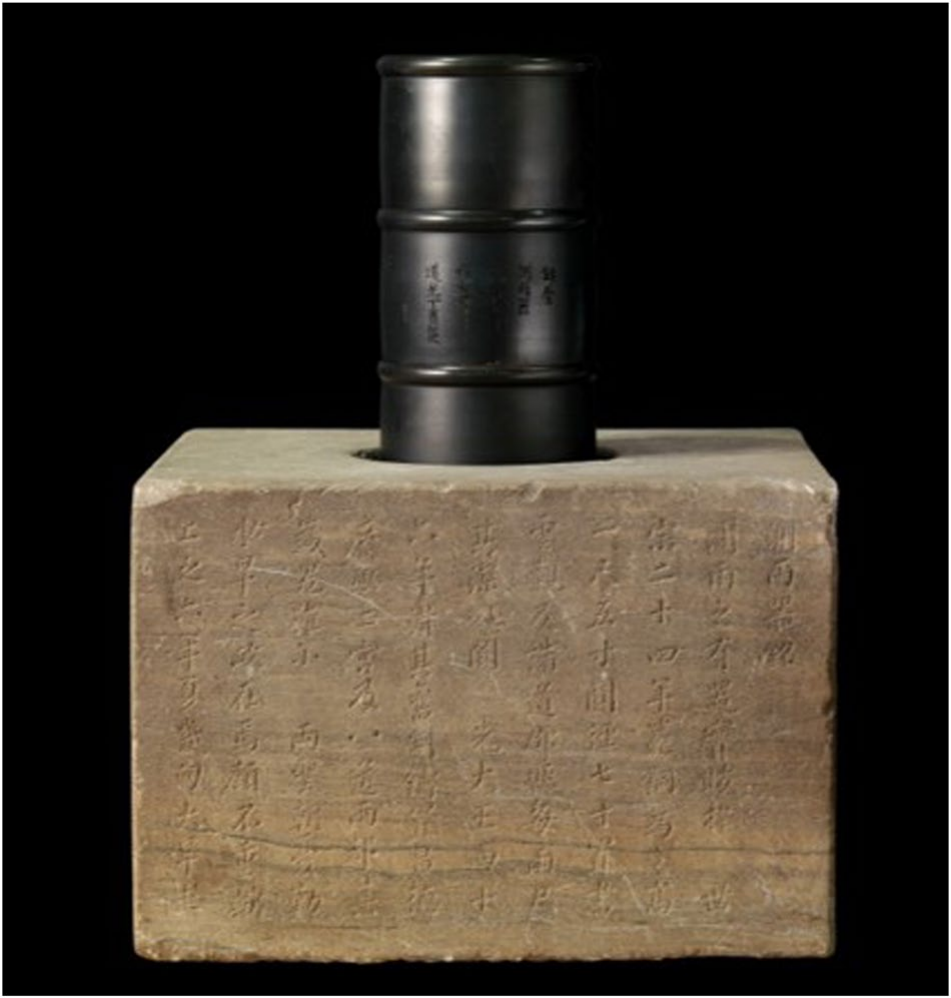
Cheugugi, the world’s oldest rain gauge devised in the Joseon Dynasty in 1441, is installed on the Cheugudae that holds the gauge.

Cheugugi was a copper device with a cylindrical shape measuring 300 mm in height and 140 mm in diameter (Fig. 1), similar to a modern tipping bucket gauge. During the Joseon Dynasty (1392–1910), the metrology system in Korea consisted of the Huang-Zhong ruler and the Zhou ruler, both adopted from Ancient China. The Zhou ruler became the primary method for national and commercial surveying in Korea, with the chi (尺) defined as the length of a finger. In the Joseon Dynasty, there were four types of measuring scales: the Zhou ruler, used for national rites and agricultural land surveys; the Yong-Jo ruler, utilized in construction, weapon production, and shipbuilding; the Cho-Rye-Gi ruler, serving for ceremonial laws and offerings; and the Po-Bak ruler, employed for measuring fabric and clothing. The historical context suggests that the Zhou ruler set the standard for the four real ruler systems^31^. There is evidence in Sejong Shillok-96^12^ records that rainfall observations were made using the Zhou ruler. One chi (尺) corresponds to a length of 200 mm, one cun (寸) is 1/10 of a chi, and one fun (分) is 1/10 of a cun.

It is noteworthy that this observation system likely underestimates both the amount and frequency of rainfall when compared to the current standard of a tipping bucket gauge, which typically has a threshold of 0.1 mm per tip^32^. Unlike the tipping bucket gauge, Cheugugi is a traditional instrument solely designed for collecting rainfall in its liquid phase only. During the Joseon Dynasty, techniques for measuring snow depth and coverage, as well as converting snow from its solid to liquid form, remained unknown. Consequently, only qualitative descriptions of snowfall events were recorded on a daily basis. It is also important to mention that in East Asia, where rainfall is primarily concentrated during the summer monsoon season, this truncation error may have little impact on rainfall measurements compared to other seasons^18,23,27^. Careful conversion is necessary because the Joseon Dynasty used unique local time and metrological units that differ from the current systems. In 1434, King Sejong invented a sundial called “Angbuilgu” and established a distinct time system based on sundial observations^33^. This time system, based on the lunar calendar, divided each day into 12 parts and initiated a new day uniformly at sunrise throughout the year, resulting in varying day lengths depending on the season^34^.

**Fig. 2:**
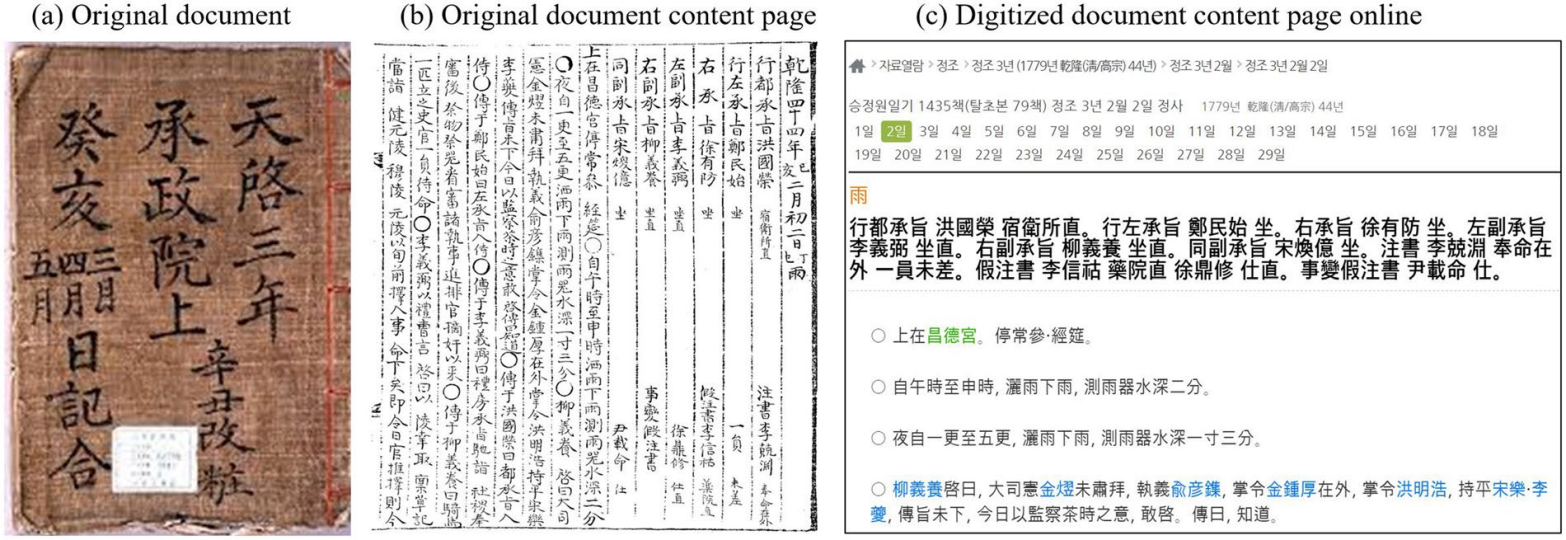
Seungjeongwon Ilgi documented daily events and weather phenomena during the 18th century Joseon Dynasty. It includes (**a**) the original document cover, (**b**) a sample page of contents dated 19 March 1779, and (**c**) the corresponding online page displaying digitized content for that specific date.

**Fig. 3:**
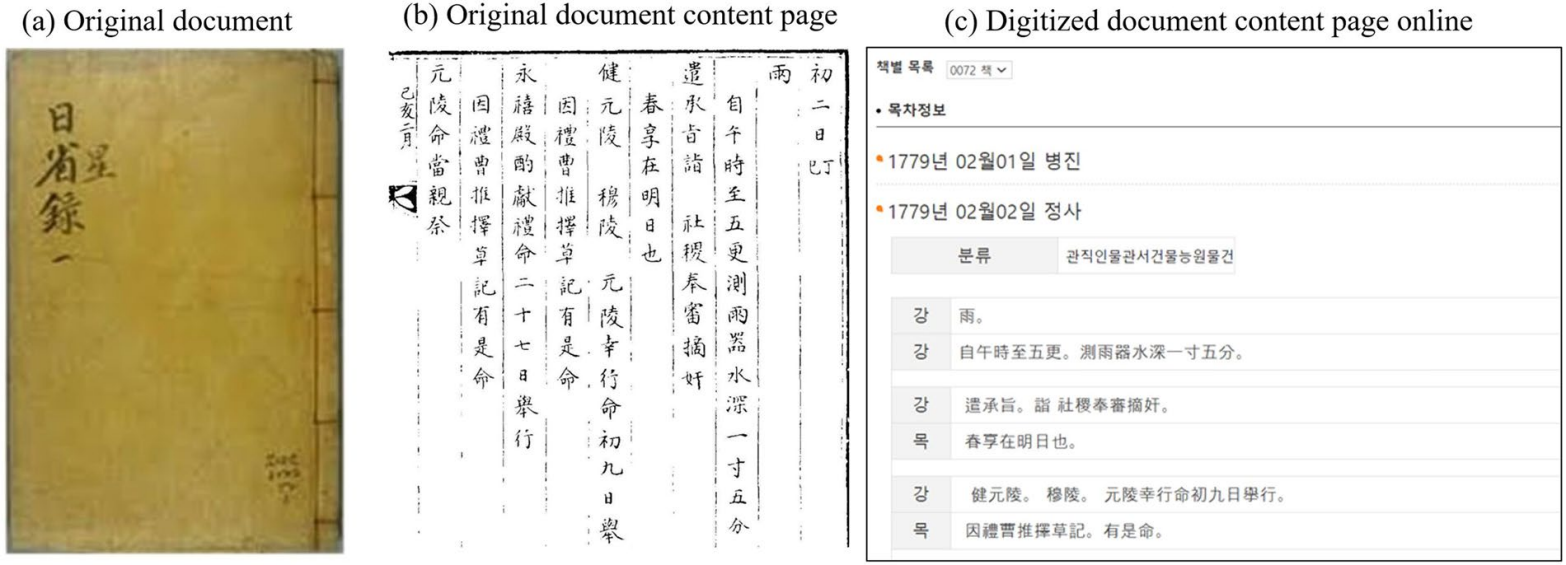
Ilseongnok documented daily events and weather phenomena during the 18th century Joseon Dynasty. It includes (**a**) the original document cover, (**b**) a sample page of contents dated 19 March 1779, and (**c**) the corresponding online page displaying digitized content for that specific date.

For this study, the date and time of rainfall events were adjusted to fit the current 24-hour system in the Gregorian calendar^35^. Given that precipitation, similar to minimum and maximum temperatures, is conventionally observed and reported in local time^36^, the time conversion in this study was adjusted to the local time of Seoul, South Korea (i.e., KST). To convert the time, we used the nautical twilight data provided by the Korea Astronomy and Space Science Institute^37^. However, for simplicity, we set the time of sunrise at 5 am based on Ahn and Park^34^. This simplification did not introduce significant bias, as most of the rainfall occurred during the summer when sunrise typically varied between 4 and 6 am based on nautical twilight in Seoul. Figures 2, 3 display the daily records for February 2nd in the lunar calendar, which corresponds to March 19th in the Gregorian calendar, during the third year of King Jeongjo in 1779. According to Seungjeongwon Ilgi (SJW hereafter), there was drizzle and rain from 11 to 17 o’clock during the daytime, totaling 2 fun. The drizzle and rain continued from 19 to 5 o’clock the next day before sunrise, totaling 1 cun and 3 fun. This is equivalent to 4 mm of rain during the daytime and 26 mm during the nighttime, resulting in a total of 30 mm. Ilseongnok (ISN hereafter) also recorded rainfall from 11 to 5 o’clock the next day, with a total of 1 cun and 5 fun, equivalent to 30 mm. Originally, the documents considered the rainfall event to be one day. However, when converting to the modern time system, the rainfall event extended to the next day. To calculate daily rainfall following the modern time system, we proportionally divided the total rainfall amount into two days based on the duration of hours.

**Fig. 4:**
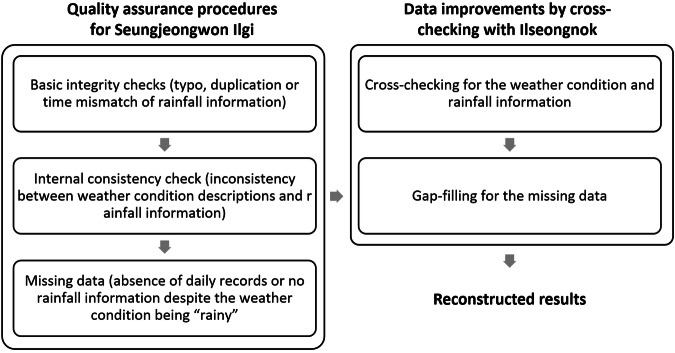
Quality assurance procedures involved basic integrity and internal consistency checks with Seungjeongwon Ilgi, and cross-checking and gap-filling were done with Ilseongnok.

### Categorization of daily weather conditions

SJW used over a hundred different word expressions to document daily weather conditions. For this study, we categorized these expressions into four primary weather conditions: clear, cloudy, rainy, and snowy. Some expressions combined conditions, such as ‘clear or cloudy’ and ‘cloudy and rain’. In such cases, we assigned priority to the four conditions in the following order: clear, cloudy, rainy, and snowy from the lowest to the highest, ultimately determining one representative condition for each day.

### Quality assurance

In historical weather data, encountering abnormal values, inconsistencies among datasets, and errors are more likely to occur^38–40^. In this study, we conducted quality assurance procedures, which involved basic integrity checks to identify erroneous editing, duplicate entries, and mis-entries^38^. We also aimed to improve accuracy and completeness by cross-checking with SJW and ISN. Initially, our quality assurance procedures focused on conducting basic integrity and internal consistency checks with SJW. Subsequently, we proceeded with cross-checking using ISN. The results of each quality assurance process are summarized in Table 1.

**Table 1:** Quality assurance (QA) and its related flags in the precipitation data.

QA process	Flag	Identified errors	Number of days
1. Basic integrity and internal consistency checks with Seungjeongwon Ilgi (SJW) only	a	Misspelling	26
	b	Overlapping, duplication, time mismatch checking	27
	c	missing daily records	444
	d	missing quantitative rainfall information	1565
	e	Inconsistency between weather condition and rainfall amount	1498
2. Adoption of Ilseongnok (ISN) records	i	Replacement with ISN when the rainfall amount was greater than 0 mm in ISN while there was no rainfall or rainfall amount of 0 mm (i.e., trace) recorded in SJW	515
	j	Replacement with ISN when the starting and ending times of rainfall events or rainfall amounts mismatch between SJW and ISN records	98

During the basic integrity check using SJW, we identified 26 typos in the weather condition descriptions, 27 instances of overlapping, duplication or time mismatch of rainfall records. Through an internal consistency check, we found 1498 instances of inconsistency between weather condition descriptions and rainfall information. Additionally, we discovered 2009 days with missing records in SJW, including 444 days without any daily records and 1565 days without rainfall amount records. Overall, there were 3560 days with erroneous or missing data, accounting for 7.5% of the entire data period (47,480 days spanning 130 years).

In the next step, we compared SJW and ISN for inconsistencies and opted to use the corresponding values from ISN. We found 98 days with inconsistent rainfall information between the two documents and 515 days with disparities in the starting and ending times of rainfall events. Figure 4 illustrates these entire quality assurance processes. Consequently, we replaced the rainfall information from SJW with ISN records for 613 days, and we filled missing values with ISN records for 2009 days. Through this cross-checking process and subsequent gap-filling, we were able to achieve completeness in our reconstructed data for the entire data period.

## Data Records

The dataset is available at zenodo 10.5281/zenodo.11076768^41^. The original and reconstructed precipitation data, along with weather conditions, have been organized into Microsoft Excel spreadsheets. For convenience, each of the daily rainfall amount and the monthly rainfall amount and frequency of precipitation days are provided as netCDF files in the same data repository along with the Excel file. The reconstructed precipitation dataset in Excel sheets provides information on rainfall amounts and frequency, as well as simple weather descriptions, accompanied by quality flags. To ensure clarity, this paper refers to precipitation as encompassing both the quantitative rainfall data and the qualitative descriptions of rain and snow.

The Excel file comprises three spreadsheets. The first sheet, named ‘daily data’, contains the daily rainfall amounts and weather codes. The second sheet, named ‘monthly data’, presents the monthly accumulated rainfall amounts and the number of rainy and snowy days for each month. The third sheet lists the weather conditions and provides a summary of their occurrences.

Table 2 presents the column labels and their corresponding descriptions for the first two data sheets. The column labeled ‘yy’ represents the year, ‘mm’ the month, and ‘dd’ the day. The simple weather descriptions are listed in the column labeled ‘wx’, written in classic Chinese letters as parsed from the digitized version of SJW. There were 123 different word expressions describing the daily weather conditions. We assigned numbers from 1 to 123 to these words and converted the Chinese characters into digits, which are listed in the ‘wx_code’ column. To simplify, we categorized these weather conditions into four states: clear, cloudy, rainy, and snowy, with corresponding codes 1, 3, 7, and 8, respectively. These codes are listed in the ‘swx’ column for SJW records and ‘rwx’ for our reconstructed data. In cases when rainfall amounts or daily records are not available, the code is set to 0. These simple descriptions are summarized in the third, “weather code,” for the entire data period.

**Table 2:** Information in the first and second sheets of the data file “Seoul_precipitation_1778-1907_Lee_etal_2024.xlsx”.

Column label	Description (SJW for Seungjeongwon Ilgi, ISN for Ilseongnok)
yy	year
mm	month
dd	day
wx	original weather description in classic Chinese letters (123 in total)
wx_code	digital codes corresponding to weather conditions (1 to 123)
swx	new categories (1, 3, 7, 8, respectively for clear, cloudy, rainy, snowy, and 0 for missing values) converted from wx_code of SJW
srn	quantitative rainfall amount in mm from SJW
rwx	new category (1, 3, 7, 8, corresponding to clear, cloudy, rainy, snowy conditions) primarily derived from SJW records and supplemented with ISN records to fill gaps
rrn	quantitative rainfall amounts in mm primarily extracted from SJW records and supplemented with ISN records
rfq	number of precipitation days, including both rainy and snowy days, obtained from rwx and rrn for the month
flag_a-d	cases of basic intrinsic error check and correction with SJW only
flag_e	cases of inconsistency check and correction between weather conditions and rainfall amount in SJW
flag_i-j	cases of adopted ISN records when the rainfall information with amount and time are inconsistent between SJW and ISN

The daily rainfall amount is recorded in the columns ‘srn,’ parsed from the SJW, and ‘rrn,’ reconstructed for this study, with the unit in millimeters. The results of the quality assurance processes are also presented in the last three columns. Through the quality assurance processes, we tagged each process result as ‘a’ for typos in daily weather, ‘b’ for cases of overlapping or re-copying of rainfall records, ‘c’ for missing daily records in the SJW document, ‘d’ for missing quantitative rainfall amount record, ‘e’ for instances of inconsistent weather descriptions and rainfall information in the daily records of SJW, ‘i’ for instances of inconsistent rainfall information between SJW and ISN, and ‘j’ for instances of inconsistent timing of rainfall events between SJW and ISN. The results tagged ‘a’ through ‘d’ are listed in the column labelled ‘flag a-d’, ‘e’ in the column labeled ‘flag e’, and ‘i’ and ‘j’ together in the column labeled ‘flag i-j’. The flags ‘a’ through ‘d’ resulted from the basic integrity checks and the flag ‘e’ from the internal consistency checks with SJW, while the flags ‘i’ and ‘j’ are from the cross-checking with SJW and ISN.

## Technical Validation

Our final dataset, referred to as R-Data in this section, is the world’s longest daily rainfall data for 130 years before the Industrial Revolution. It has undergone all quality assurance procedures and has no missing values. We analyzed the monthly time series of R-Data to assess its suitability as climate data. According to the z-score applied to the Kolmogorov-Smirnov test, the monthly rainfall time series in R-Data conforms to a normal distribution^42^. The highest monthly rainfall occurred in July 1832, reaching 1296 mm, while the peak summertime rainfall was 2022 mm from June to August in 1821. The mean annual rainfall is 1200 mm, with an interannual standard deviation of 386 mm. Notably, this mean value is approximately 15% less than the present climate value of 1418 mm (1991–2020) for Seoul, Korea.

We compared the monthly rainfall data in R-Data with data reported in previous studies. To facilitate this comparison, we manually compiled data from image files of the previous studies. This compilation included daily rainfall data from Jhun and Moon^22^, referred to as J-Data, sourced exclusively from the SJW. We also included monthly rainfall data from Wada^18^, referred to as W-Data, extracted from the Joseon ancient observation record report at the Japanese government general of Korea during the Japanese colonial era. In the next paragraph, we refer to the SJW records as S-Data, which underwent our quality assurance processes before cross-checking with ISN for a feasible comparison with J-Data.

In J-Data, there were 7920 days with daily rainfall exceeding 0 mm, while in S-Data, there were 8087 days. Notably, there were inconsistencies in 5394 days between these two datasets. These inconsistencies were generated by (1) rainfall events on two consecutive days (3545 days), (2) missing data in J-Data (1180 days), and (3) wrong values in J-Data compared to S-Data (669 days). We speculate that data were omitted and mistreated during manual reading before digitization. Furthermore, we found that rainfall values in J-Data had day-shifted for several months during the periods of March-August 1825 and September 1825-February 1826.

**Fig. 5:**
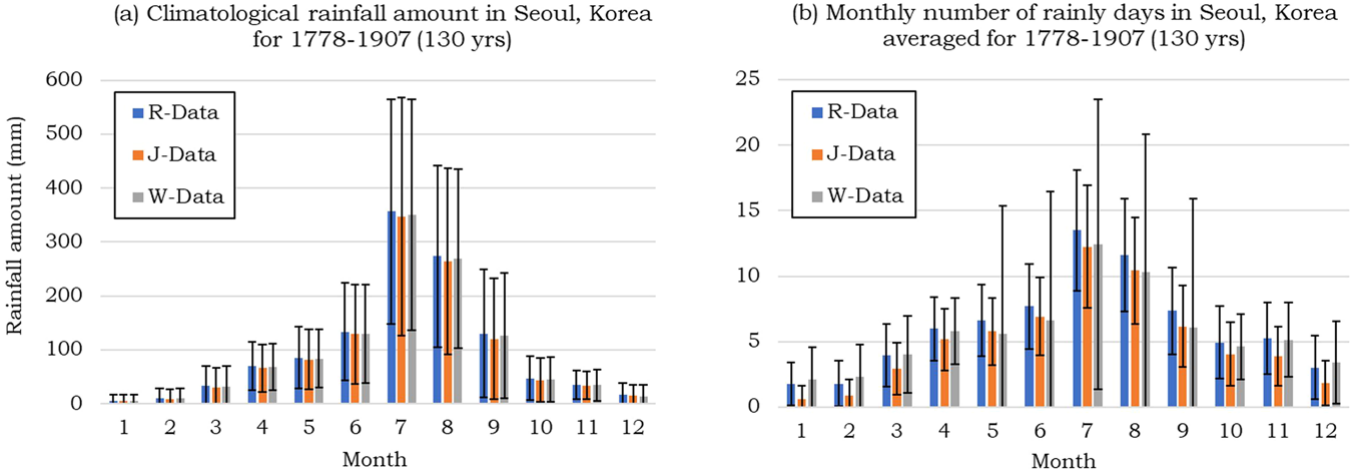
(**a**) Monthly rainfall amount and (**b**) the number of rainy days averaged from 1778 to 1907 during the Joseon Dynasty observed in Seoul, Korea. The blue color denotes the reconstructed data in this study, the orange color represents the data drawn from Jhun and Moon (1997), and the gray color represents the data drawn from Wada (1917). Error bars represent the interannual standard deviation for each month.

Figure 5 illustrates the climatological mean monthly precipitation values and the number of precipitation days involving rain and/or snow. R-Data consistently shows larger values each month compared to J- and W-Data, up to 10 mm higher in September. This difference can be attributed to the gap-filling procedure using ISN (Fig. 5a). The interannual variability in monthly rainfall is substantial across all months, with no significant differences observed in both the climatological mean and interannual standard deviation.

**Fig. 6:**
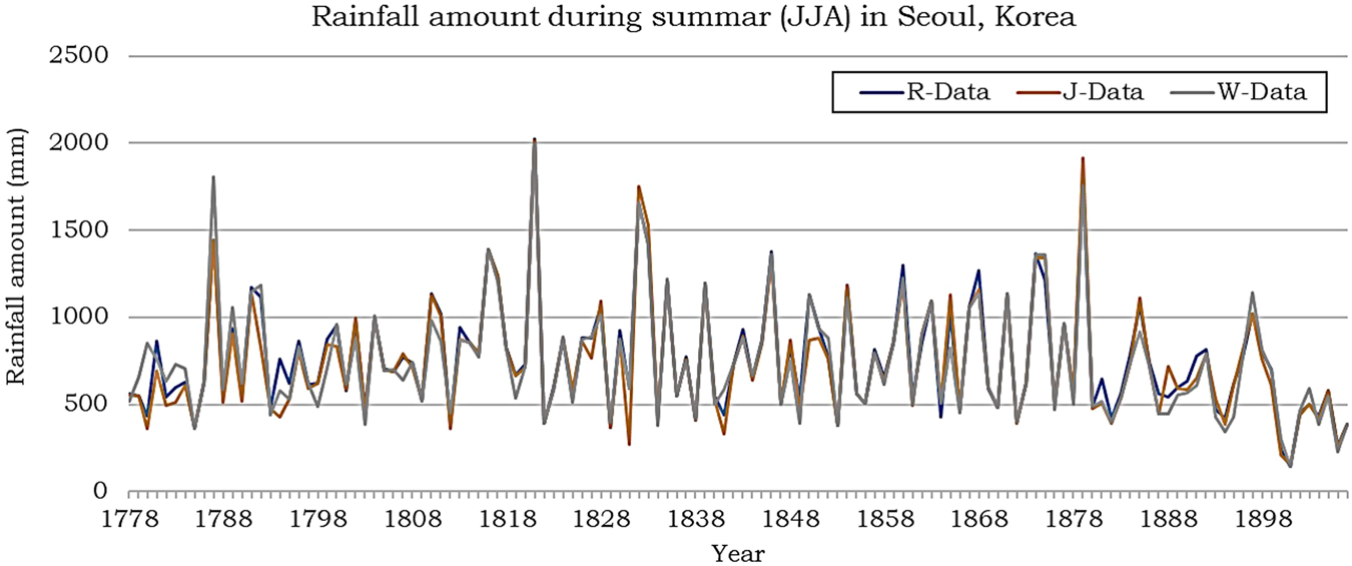
Summertime (June to August) rainfall amount from 1778 to 1907 during the Joseon Dynasty observed in Seoul, Korea. The blue color denotes the reconstructed data in this study, the orange color represents the data drawn from Jhun and Moon (1997), and the gray color represents the data drawn from Wada (1917).

The number of precipitation days in R-Data is comparable to W-Data, whereas J-Data records fewer rainy days compared to both R- and W-Data every month (Fig. 5b). R-Data exhibits more rainy days than W-Data from April to November but fewer rainy days during the colder seasons (December to March). The interannual standard deviation of W-Data for the number of rainy days is unreasonably high during warm seasons (May to September), which raises skepticism about potential errors in extracting data from image files or handling missing values. Figure 6 shows the time series of summer mean rainfall amounts derived from R-, W-, and J-Data. The general trends in the time series remain consistent across all datasets, although there are summers when the differences among the datasets exceed 100 mm, reaching a maximum disparity of 489 mm in 1780.

### Supplementary information

daily precipitation data as MS excel format

## Data Availability

The digitized SJW (in National Institute of Korean History at https://sjw.history.go.kr/main.do) and ISN (in Kyujanggak Institute for Korean Studies at https://kyudb.snu.ac.kr/search/search.do) are openly available as image and text (in Korean and Chinese) files. The precipitation data reconstructed in this study is available at 10.5281/zenodo.11076768.
